# Optimal Size of Electrocorticography Grids for Classification of Hand Movements

**DOI:** 10.1007/s12021-026-09807-z

**Published:** 2026-08-07

**Authors:** Elena C. Offenberg, Dirk Keller, Siamak Mehrkanoon, Julia Berezutskaya, Mariska J. Vansteensel, Mariana P. Branco

**Affiliations:** 1 Department of Neurology and Neurosurgery, University Medical Center Utrecht Brain Center, Utrecht University, Utrecht, The Netherlands; 2 Department of Information and Computing Sciences, Utrecht University, Utrecht, The Netherlands; 3 Donders Institute for Brain, Cognition and Behaviour, Radboud University, Nijmegen, The Netherlands

**Keywords:** Electrocorticography, Hand movement, Grid size, Classification, Brain-computer interfaces

## Abstract

Implanted Brain-Computer Interfaces (BCIs) hold significant promise as a replacement for conventional assistive technologies for people with extensive motor impairments. In recent years, significant progress has been made in enhancing BCI performance, bringing clinically viable systems within reach. Some of these developments rely on increasingly large numbers of high spatial-density electrocorticography (ECoG) electrodes, which enable the extraction of spatially detailed information from extensive brain areas, but may also elevate surgical burden, posing a challenge for the clinical adoption of implanted BCIs. To mitigate this risk, we conducted an exhaustive investigation of hand movement classification performance involving 4, 5, and 8 classes in nine individuals with epilepsy, exploring all possible rectangular ECoG subgrids within the 32-, 64-, and 128-channel grids that were implanted in these individuals. Our findings reveal that the surface area of ECoG grids can be substantially reduced by 75–94% compared to the original grids, without a meaningful decline in classification performance, if electrodes are placed over informative areas. Classification performance across datasets was stable for progressively smaller subgrids until a critical threshold of approximately 60mm^2^ was reached, below which performance declined substantially. We show that smallest subgrids with an area above the threshold achieved equally high classification F1 scores (range 81.64-99.71%) as the full grids with an area > 230mm^2^ (range 82.85-96.75%). We conclude that ECoG-based BCI can be used to accurately decode up to seven different hand movements from well-located grids with a small number of electrodes, paving the way for smaller and safer BCI implants.

**Supplementary Information:**

The online version contains supplementary material available at https://doi.org/10.1007/s12021-026-09807-z.

## Introduction

Assistive technologies are used as a solution for computer control and communication for individuals with severe motor and speech impairments due to acute events such as brainstem stroke, or neurodegenerative diseases such as amyotrophic lateral sclerosis. Operation of conventional augmentative and alternative communication devices, however, depends on reliable, residual movements, which many individuals with severe motor impairments are unable to perform. For those cases, implanted Brain-Computer Interfaces (BCIs) may offer an alternative communication channel by bypassing the non-functional motor system (Vansteensel & Jarosiewicz, 2020). BCIs extract neuroelectrical signals, typically from either the hand or mouth region, of the brain and translate them to discrete or continuous computerized commands for communication and computer access. In the last decade, advancements in BCI technology have focused on improving decoding accuracy, communication rates and assessing long-term reliability (e.g., Luo et al., 2023; Metzger et al., 2023; Card et al., 2024; Vansteensel et al., 2024; Candrea et al., 2024). In particular, high-density (HD) electrocorticography (ECoG) has been proposed as a valuable neuronal recording modality for high-performance decoders and clinically viable BCI solutions (Silva et al., 2024; Lorach et al., 2023; Natraj et al., 2025). ECoG grids measure neural signals from spatially distinct populations of neurons. The high frequency band of ECoG signals (> 40 Hz), specifically, is tightly coupled, both temporally and spatially, to changes in population firing rates in the immediate vicinity of recording electrodes (Ray et al., 2008; Ray et al., 2008a, b; Lei et al., 2026) and has been associated with movement execution (Crone et al., 1998; Miller et al., 2007). A key advantage of ECoG is the stability of signals over time, as evidenced in preclinical (Yeager et al., 2008; Chao et al., 2010; Rouse et al., 2013) and clinical trials (Lee et al., 2022; Vansteensel et al., 2016; Luo et al., 2023; Silva et al., 2024), which enables stable BCI performance over several months and years (Vansteensel et al., 2024; Pels et al., 2019; Nair et al., 2020; Wyse et al., 2024).

Recent clinical trials employing HD-ECoG grids have targeted extensive cortical coverage by implanting large or multiple grids, encompassing 128 to 253 channels (Angrick et al., 2024; Luo et al., 2023; Moses et al., 2021; Metzger et al., 2023). While the use of large numbers of HD-ECoG electrodes has delivered highly promising results in terms of decoding performance and BCI-based communication speed (Angrick et al., 2024; Metzger et al., 2023), this approach may potentially compromise implant safety and increase surgical burden. Indeed, evidence suggests that ECoG-related complications occur more frequently with larger numbers of implanted electrodes (Hamer et al., 2002; Nagahama et al., 2018). To achieve meaningful clinical impact for the populations of individuals who could benefit from BCI technologies, surgical procedures for neural recording implants should be minimally invasive, avoiding neural tissue damage as well as minimizing peri- and post-surgical complication risks and burden (Silva et al., 2024). One strategy to minimize surgical risk is to minimize the required size of the skull opening by using smaller electrode grids (e.g., Ryvlin, 2025). In earlier work, we showed that articulator and tongue movements can be distinguished from a relatively small HD-ECoG surface area (Salari et al., 2019). More recently, we assessed the effects of HD-ECoG surface area reduction on the classification accuracy of individually pronounced words (Offenberg et al., 2026), and showed that grid surface area can be decreased substantially without compromising performance. For the decoding of hand and finger movements, several studies have shown decent classification accuracy with a limited number of clinical, 1 cm resolution, electrodes (Milekovic et al., 2012; Li et al., 2017; Verwoert et al., 2021), but the relationship between classification accuracy and HD-ECoG grid size remains to be determined.

Here, we investigated the effect of decreasing the number of HD-ECoG electrodes on the performance of a hand/finger movement classifier and determined the extent to which the HD-ECoG grid surface area can be reduced while preserving classification performance of 4-, 5-, and 8-classes of hand/finger movements. To achieve this, we performed an exhaustive spatial search of subgrids within the original grids implanted over the sensorimotor cortex of nine able-bodied individuals.

## Materials and Methods

### Participants

Data were collected from nine participants with epilepsy who received a subchronic implantation with clinical ECoG grids for diagnostic purposes (Table 1). For research purposes, an additional HD electrode grid was placed over a clinically non-relevant area of the precentral and/or postcentral gyrus of the sensorimotor cortex. The exact placement of the grids was identified by co-registering the pre-operative T1-weighted magnetic resonance imaging (MRI) scan (Philips 3T Achieva, Best, the Netherlands) with a post-operative computed tomography (CT; Philips Tomoscan SR7000, Best, the Netherlands) scan and was subsequently mapped to the 3D surface of the participants pre-operative MRI scan (Hermes et al., 2010; Branco et al., 2018). This study adhered to the Declaration of Helsinki (2024) and received ethics approval from the University Medical Center Utrecht ethics committee. All participants provided written informed consent for the additional placement of a HD-ECoG grid and their participation in the research.

**Table 1:** Participant, grid and task details. Task performed per participant, as well as number of trials (#Trials) per active condition (A) and in total (A + R, active+rest), the number of channels (#Channels) included in the analysis (I) and the total number of implanted HD-ECoG electrodes (T), the inter-electrode distance (IED, in mm) and exposed electrode diameter (ED, in mm). Hemisphere – targeted hemisphere implanted with HD-ECoG

Sub-ID	Task	#Trials		#Channels		Age	Sex	Moved hand	Handedness	Hemisphere	Grid Manufacturer	Grid dimension	Grid IED	Grid ED
		A	A + *R*	I	T									
01	Gesture Finger	36 85	73 151	32	32	19	Female	Right	Right	Left	AdTech	4 × 8	3	1.3
02	Gesture	68	138	59	64	45	Female	Left	Left	Right	AdTech	8 × 8	3	1.3
03	Gesture	36	71	29	32	29	Male	Right	Right	Left	AdTech	4 × 8	3	1.3
04	Gesture Finger	34 90	69 180	31	32	19	Male	Right	Right	Left	AdTech	4 × 8	3	1.3
05	Gesture Finger	34 88	69 173	32	32	42	Male	Right	Right	Left	AdTech	4 × 8	3	1.3
06	Finger	89	179	123	128	30	Female	Left	Right	Right	PMT	16 × 8	3	1
07	Finger	89	179	118	128	50	Female	Left	Right	Right	PMT	16 × 8	3	1
08	Finger	90	152	64	64	20	Female	Right	Right	Left	PMT	8 × 8	4	1
09	Finger	84	164	128	128	36	Female	Right	Right	Left	AdTech	16 × 8	4	1.17

### Data Acquisition

The HD-ECoG grids consisted of 32, 64, or 128 electrodes arranged in a lattice formation. The platinum-iridium disc electrodes had an exposed diameter of 1 to 1.3 mm and an inter-electrode spacing of 3–4 mm, and were embedded in silicone sheets (AdTech, Racine, USA; or PMT Corporation, Chanhassen, MN, USA; Table 1). Neural signal recording for sub-01 to sub-05 was performed using a 128-channel Micromed LTM system (Treviso, Italy; 22 bits) with a hardware bandpass filter of 0.15–134.4 Hz and a sampling frequency of 512 Hz. For sub-06 to sub-09, a Blackrock system (Microsystems LLC, Salt Lake City, USA) with a digital bandpass filter of 0.3–500 Hz and a sampling frequency of 2000 Hz was used.

### Task Description

This study integrated data from prior publications (Siero et al., 2014; Bleichner et al., 2016; Verwoert et al., 2021). The first task involved finger flexion of the index finger, little finger, and thumb (Finger task), while the second task involved hand gesture formations associated with the American Sign Language letters D, F, V, and Y (Gesture task). Both tasks employed an event-related design with interleaved hand movement conditions. Participants were instructed to initiate movements based on visual cues. For the Finger task, participants (sub-01 and sub-04 to sub-09; *N* = 7) performed one or two finger flexions immediately after cue onset, then returned to a resting position. This design included 30 trials for each of the three fingers, with an intertrial interval of 4.4 s for sub-01, sub-04 and sub-05, and 5 s for remaining subjects. For the Gesture task, participants (sub-01 to sub-05; *N* = 5) performed a cued gesture and maintained the hand posture until the end of the trial (6 s) before returning to rest (6 s). Each participant performed 10 trials per each of the 4 gestures. The tasks were performed with the hand contralateral to the grid location, with one participant (sub-02) performing the Gesture task twice. For this participant and task, the raw data was concatenated into one run before signal preprocessing, yielding (maximum) 20 trials per gesture. Hand movements were recorded with a dataglove (5DT, Irvine, CA, USA, 20ms sampling time). These recordings were used to extract movement onset (Branco et al., 2017). HD-ECoG data was subsequently aligned to movement onset and organized in the BIDS standard format (Holdgraf et al., 2019).

### Dataset Formation

In total, four datasets were constructed from the Finger and Gesture task data. The Finger and Gesture task data as collected correspond to a dataset with three fingers and rest (4-class Fingers) and a dataset with four gestures and rest (5-class Gestures). To compare performance between gestures and fingers, we created an additional Gesture dataset with only three gestures and rest (4-class Gestures) by excluding the poorest-performing gesture (Gesture D) in a binary Support Vector Machine classifier against rest. Last, a dataset including three fingers, four gestures and rest (8-class Hand) was created by combining data from the Fingers and Gesture tasks.

### Preprocessing

Preprocessing was conducted using Python (v3.9) and the MNE library (v1.16). Flat or noisy electrodes, as identified earlier (Siero et al., 2014; Bleichner et al., 2016; Branco et al., 2017; Verwoert et al., 2021) were removed from analyses to eliminate noise sources (24 Hz hardware noise and 50 Hz line noise and their harmonics). The data was re-referenced using common average referencing (excluding flat or noisy channels). Data sampled at 2000 Hz was downsampled to 512 Hz for consistency across participants after applying a finite impulse response bandpass filter with a Hamming window for the frequency range of 60–130 Hz. These steps were applied once during preprocessing and remained the same for all subsequent analysis. For classifying individual finger and gesture movements, we used segmentation windows of W_Fingers_ = [-0.5, 1.5]s and W_Gestures_ = [-0.5, 2.5]s, where time zero corresponds to movement onset. The gesture‑related window was extended to account for the slower behavioral and neural dynamics associated with hand gestures compared with single‑finger flexion. Rest epochs were extracted from intertrial intervals for the Finger task or from cued rest periods for the Gesture task, and were cropped to ensure non‑overlapping segments that matched the duration of active‑trial windows: R_Fingers_ = [2, 4]s relative to finger movement onset and R_Gestures_ = [1.5, 4.5]s relative to rest cues. Active and rest epochs with dataglove amplitude two standard deviations larger than the mode of the amplitude (abnormal movement) or where the participant performed the wrong movement (as confirmed by visual inspection of the dataglove data) were removed from further analysis. For classification of all hand movements (combination of both tasks) and rest (8-class Hand), a different segmentation window of W_Hand_ = [-0.5, 2 s] was used for both finger movements and gestures, to create a common feature vector with the same temporal dimension for 8-class classification, striking a compromise between both tasks by adding more rest to the segmentation window of the Finger task (0.5s) and removing some active trial data from the Gesture task (-0.5s). Because both tasks were recorded at different times per participant, and to avoid training the classifier on two types of rest, we chose to only use the rest trials from the Gesture task for the 8-class classification. For all tasks, a continuous Morlet Wavelet transformation extracted spectral power features for the high-frequency band (60–130 Hz) in 2 Hz bins. To reduce the feature space, the spectral dimension was averaged, and the time dimension was decimated to 50 Hz. The resulting spectral power feature vector was used for subsequent model training.

### Classification

The spectral power of the spatio-temporal feature vector (with dimensions corresponding to the grid width, grid height, and number of time points) was scaled into a 0–1 range using a min-max scaler implemented within the classification pipeline, ensuring that scaling parameters were learned from the training data within each cross-validation fold and applied to the corresponding test data prior to classification. A Support Vector Machine (SVM) with a linear kernel and default hyperparameters (SVC(kernel="linear")), chosen for its demonstrated effectiveness for this type of classification problems (e.g., Yanagisawa et al., 2009, 2011; Elgharabawy & Wahed, 2016; Keller et al., 2024; Offenberg et al., 2026), was trained on each dataset separately. Performance was evaluated using a stratified nested 10-fold cross-validation procedure. Within each outer training fold, an inner cross-validation loop was used to select the optimal subgrid configuration. The final model was then evaluated on the corresponding held-out outer test fold, and all reported performance metrics reflect these outer-loop test results. Classification performance was evaluated using a weighted F1 score to account for class imbalance. All analyses were implemented in Python 3.9, using scikit-learn (v1.4.0). The decoder’s baseline performance (hereafter referred to as ‘chance level’) was determined based on the majority class prediction across participants, which depends on the label distributions. The majority class of an imbalanced dataset (more rest than active trials) refers to the prediction of the class with most trials (rest class) for every observation. As a conservative ‘chance level’, we used the highest baseline F1 score across participants for a given dataset.

### Exhaustive Subgrid Search

A spatially constrained exhaustive search over the entire grid space was conducted to assess performance distribution among all rectangular ‘subgrids’ within the original HD-ECoG grid. The search began with a single electrode configuration (1 × 1) and progressed iteratively through all possible subgrid sizes (i.e., 1 × 2, 2 × 1, 2 × 2, 1 × 3, etc.) up to the full grid size (4 × 8, 8 × 8, or 16 × 8). The total number of possible rectangular subgrids for the 32-, 64-, and 128-electrode grids was 360, 1296, and 4896, respectively, as for a given grid of size W × H, the number of possible rectangular subgrids (T_g_) is defined by all combinations of start and end points for number of electrodes *e* in the columns W and rows H:

1 $$\:{T}_{g}\text{}\mathrm{=}\text{}\frac{{\mathrm{W}}_{\mathrm{e}}({\mathrm{W}}_{\mathrm{e}}+1)}{2}\cdot\:\frac{{\mathrm{H}}_{\mathrm{e}}\left({\mathrm{H}}_{\mathrm{e}}+1\right)}{2}$$

where W_e_ and H_e_ are the number of electrodes in the width (W) and height (H) of the grid, respectively. For each subgrid configuration, the SVM model was trained and evaluated, and the F1 scores were recorded. The exhaustive search was repeated with 20 different random seeds to initialize pseudo-random number generation in model initialization, stochastic processes in the SMO solver, and the generation of cross-validation splits (including fold assignment and sample ordering). For each subgrid, performance was averaged across all folds and all seeds to reduce variance due to fold assignment, model initialization and optimization.

### Performance-area Trade-off

To identify optimal grid configurations, balancing performance and surface area from the exhaustive search results, performance area trade-off curves were generated to visualize the relationship between grid area (in mm^2^) and performance (F1 score in %). The surface area SA of a considered subgrid g was calculated as follows:

2 $$\:S{A}_{g}\mathrm{=}{\prod\:}_{i\mathrm{=}1}^{2}({a}_{i}\cdot\:IE{D}_{g}\mathrm{+}E{D}_{g})$$

With coefficient a_i_ defined as

3 $$\:{a}_{i}\mathrm{=}\left\{\begin{array}{c}\left({W}_{e}\mathrm{-}1\right),\text{}\text{}\mathrm{if}\text{ }i\mathrm{=}1\\\:\left({H}_{e}\mathrm{-}1\right),\text{}\text{}\mathrm{if}\text{ }i\text{}\mathrm{=}2\end{array}\right.$$

where SA_g_ refers to the surface area of the considered subgrid g, W_e_ and H_e_ denote the number of electrodes e in the width (W) and height (H) of the subgrid g, IED represents the inter-electrode distance, and ED signifies the electrode diameter of electrodes in the grid g.

### Informative Electrodes

To investigate the spatial distribution of the most informative electrodes (I_e_), we chose an averaging approach for each channel, where the importance value of each electrode was calculated by averaging the F1 scores of all subgrids containing the specific electrode (N_e_):

4 $$\:{I}_{e}\mathrm{=}\frac{1}{{N}_{e}}{\sum\:}_{i\mathrm{=}1}^{{N}_{e}}F{1}_{i}$$

I_e_ is the importance value of electrode e, N_e_ is the number of subgrids that include electrode e, and F1_i_ is the F1 score of the i-th subgrid containing electrode e. Therefore, each electrode’s importance reflects the performance of all subgrids that include it.

### Smallest Performing Subgrids

To identify the smallest subgrids that maintain high performance, we performed non-inferiority tests for each dataset and participant. Non-inferiority was assessed across stochastic realizations (random seeds), treating each seed as a paired observation across grid sizes. A non-inferiority margin of Δ = 0.10 was defined a priori. For each grid size, a one-sided bootstrap confidence interval of the performance difference relative to the full grid was computed. A grid size was considered non-inferior if the lower bound of the confidence interval exceeded − Δ. For each participant, the critical point was the largest grid size that did not satisfy the non-inferiority criterion. We then selected the largest critical point value across participants and datasets as a conservative, global threshold (t = 57.19 mm^2^). Last, we identified the 10 smallest subgrids above the global threshold per participant and dataset, and identified their position, area and F1 score.

## Results

### Classification Performance

Classifier performance across all seeds, participants and datasets revealed that the SVM trained on the full grid resulted in above chance level classification (Table 2). Moreover, the best-performing subgrids (from the exhaustive subgrid search) performed in the same range as full grids across datasets (classification F1 score 82.85–96.76% for full grids compared with average 83.55–96.43% for best subgrids), Table 2).

**Table 2:** Global performance. Comparison of chance, full, and best subgrid classification performance (F1 score in %) for each dataset (mean and standard deviation, std, across participants). The 4-class Gesture dataset included Gesture F, Y, V and rest. Results are based on the mean score over cross-validation folds for each participant. Chance level was computed by a majority label prediction on the original grid

	Chance Mean ± Std	Full grid Mean ± Std	Best subgrid Mean ± Std
4-class Fingers	31.05 ± 3.66	96.76 ± 2.47	96.43 ± 1.80
4-class Gestures	41.44 ± 2.08	90.83 ± 4.77	86.29 ± 6.28
5-class Gestures	34.15 ± 0.64	82.85 ± 5.80	83.55 ± 6.82
8-class Hand	8.22 ± 0.31	90.61 ± 6.21	89.73 ± 3.17

### Performance-area Analysis

**Fig. 1:**
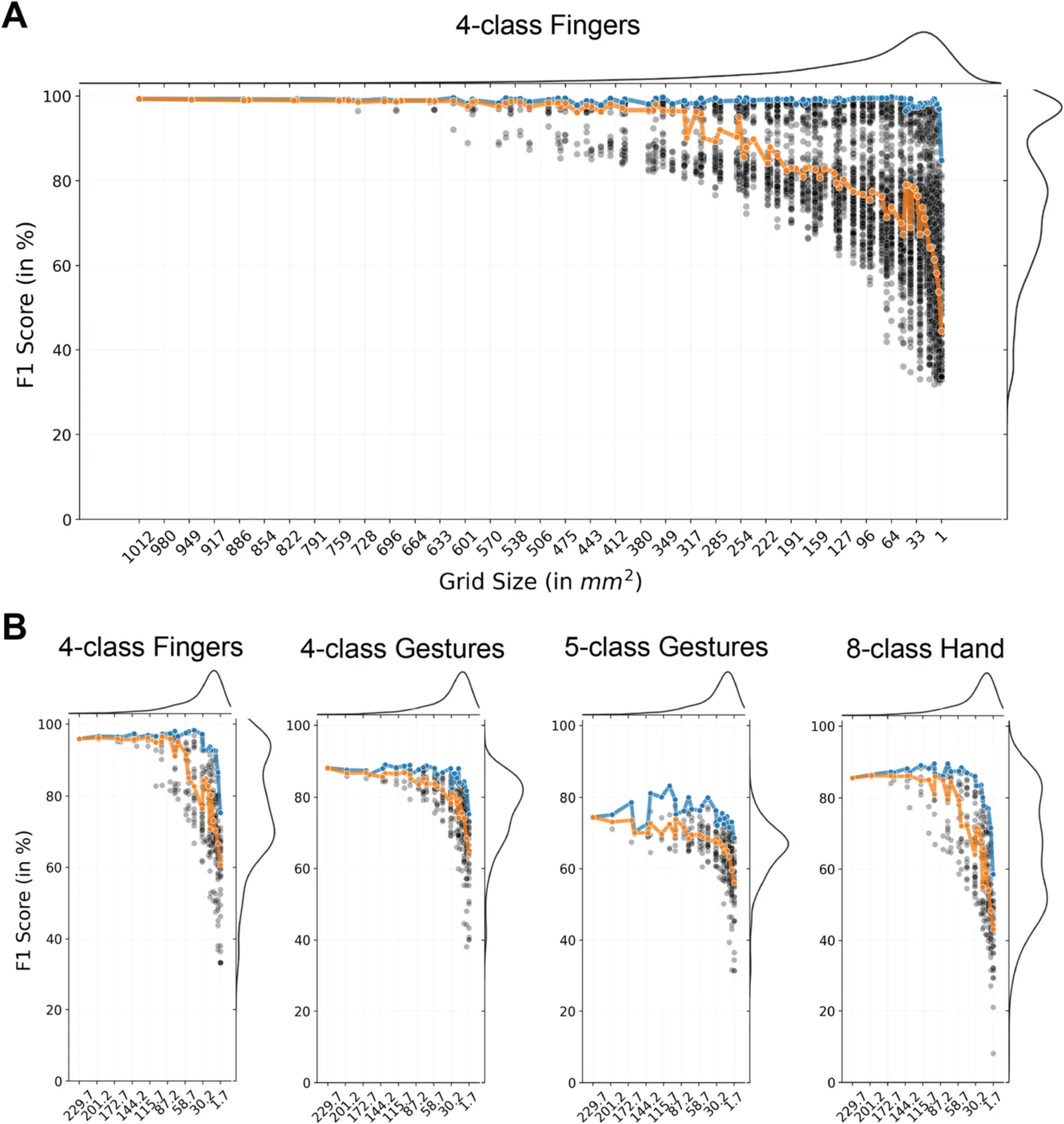
Example of the Performance-Area curve. F1 scores for two individual participants as a function of grid size (surface area in mm^2^). (**A**) 4 classes Finger dataset for sub-06 who was implanted with a 16 × 8 electrode grid and (**B**) 4-class Finger dataset, 4-class Gesture dataset, 5-class Gesture dataset, and 8-class Hand dataset for sub-04 starting from the full grid of 8 × 4 electrodes. Each panel depicts the Performance-Area curve, showing the change in performance when decreasing the original grid surface area (far left) towards the minimum possible grid size of 1 × 1 electrode (far right) for best subgrids (blue) and randomly placed subgrids (median performance in orange) over the sensorimotor cortex

**Fig. 2:**
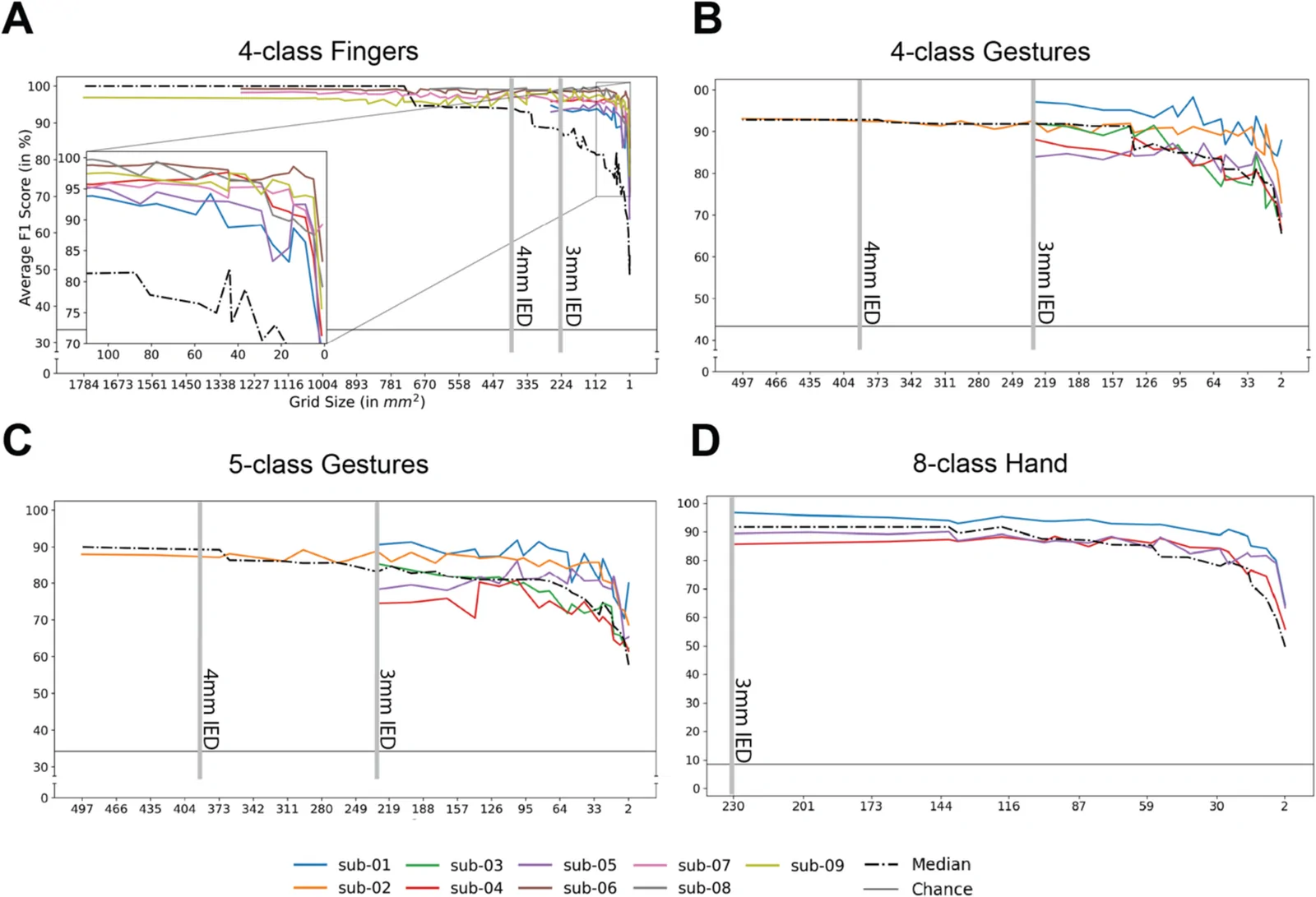
Performance-Area curve across participants. F1 score for all participants for (**A**) 4-class Finger dataset, (**B**) 4-class Gesture dataset, (**C**) 5-class Gesture dataset, and (**D**) 8-class Hand dataset. Each panel of a given dataset represents the change in performance of the best subgrids across different grid sizes (surface area in mm^2^, from largest on the left to smallest on the right) for each participant (colored lines). In addition, each panel shows the median over all subgrids and participants for a given grid size (dash-dotted black lines). Vertical gray lines indicate the surface area of a 4 × 8 grid with 3 mm and 4 mm IED (both with 1 mm ED), respectively. The horizontal gray line indicates the highest chance level across all participants (majority label). Inter-Electrode Distance, IED; Electrode Diameter, ED

The relationship between classification performance and surface area generally followed a hyperbolic trend, with a clear decline only for small surface areas (Fig. 1 for one example participant and Fig. 2 for all participants). Classification performance showed a sharper decline for best subgrids and more gradually for randomly placed grids (median line) (Figs. 1 and 2). Randomly placed grids were used as a proxy for the expected performance of subgrids positioned over the sensorimotor cortex without any prior knowledge of the underlying informative regions. We observed considerable variation in classification performance among subgrids, with suboptimal performance occurring more frequently in smaller subgrids (Fig. 1). Interestingly, performance in some participants briefly increased with area reduction before it sharply decreased (see for example sub-04 in Fig. 1B).

Combining both tasks in a single analysis (8-class Hand) confirmed that the relationship between grid size and classifier performance was consistent across participants, hand movements, task complexity and electrode spacing and diameter (Fig. 2). The relationship between classification performance and surface area was stable from full-grid size to the critical point, after which it sharply decreased. This critical point ranged between 9.49 mm^2^ and 57.19 mm^2^ across participants and datasets (Table 3). Considering the upper limit of this range (57mm^2^) as a conservative threshold, our results suggest that the surface area can be reduced by at least 75% from its original size for the smallest 32-channel grids (IED of 3 mm ED of 1.3 mm, approximate area 230mm^2^) and by up to 94% for the largest 128-channel grids (IED of 3 mm ED of 1.3 mm, approximate area 1012mm^2^) without relevant performance loss. The threshold of 57mm^2^ is equivalent to an electrode grid with approximately 12 (4 × 3, 54mm^2^) electrodes at 3 mm IED and 9 (3 × 3, 64mm^2^) electrodes at 4 mm IED.

**Table 3:** Critical points. Critical points (in mm²) derived from non-inferiority tests for each dataset and participant. The table reports the smallest subgrid with area > 57 mm² and its corresponding F1 score. When multiple subgrids met this criterion with the same area, the subgrid with the highest F1 score was selected. The table also includes the best-performing subgrid from the exhaustive subgrid search, including the full grid when applicable, and its F1 score are also shown

Dataset	Participant	Critical points (mm^2^)	Smallest subgrid area > 57mm^2^ (mm^2^)	F1 score smallest grid > 57mm^2^ (%)	Best subgrid area (mm^2^)	Best subgrid F1 score
4-Class-fingers	sub-01	8.89	70.09	93.75	106.09	95.35
4-Class-fingers	sub-04	8.89	70.09	96.07	75.19	98.02
4-Class-fingers	sub-05	5.12	70.09	93.28	75.19	95.87
4-Class-fingers	sub-06	14.39	64.0	99.71	64.0	99.71
4-Class-fingers	sub-07	14.39	64.0	97.57	598.0	99.18
4-Class-fingers	sub-08	29.23	65.0	98.56	169.0	99.76
4-Class-fingers	sub-09	14.39	57.53	97.47	274.89	99.41
4-Class-gestures	sub-01	17.29	70.09	98.41	82.99	99.72
4-Class-gestures	sub-02	16.20	70.09	93.08	70.09	93.08
4-Class-gestures	sub-03	57.19	70.09	86.16	118.99	95.01
4-Class-gestures	sub-04	28.84	70.09	88.80	136.99	88.58
4-Class-gestures	sub-05	9.49	70.09	84.80	106.09	88.96
5-Class-gestures	sub-01	28.38	70.09	92.50	95.89	93.49
5-Class-gestures	sub-02	15.25	70.09	87.31	167.89	89.83
5-Class-gestures	sub-03	57.19	70.09	81.64	198.79	85.14
5-Class-gestures	sub-04	17.29	70.09	76.72	106.09	83.18
5-Class-gestures	sub-05	9.49	70.09	83.60	106.09	87.57
8-Class-hand	sub-01	28.87	70.09	95.02	229.69	96.44
8-Class-hand	sub-04	28.87	70.09	87.38	118.99	89.57
8-Class-hand	sub-05	9.49	70.09	87.15	75.19	89.98

### Channel Importance

**Fig. 3:**
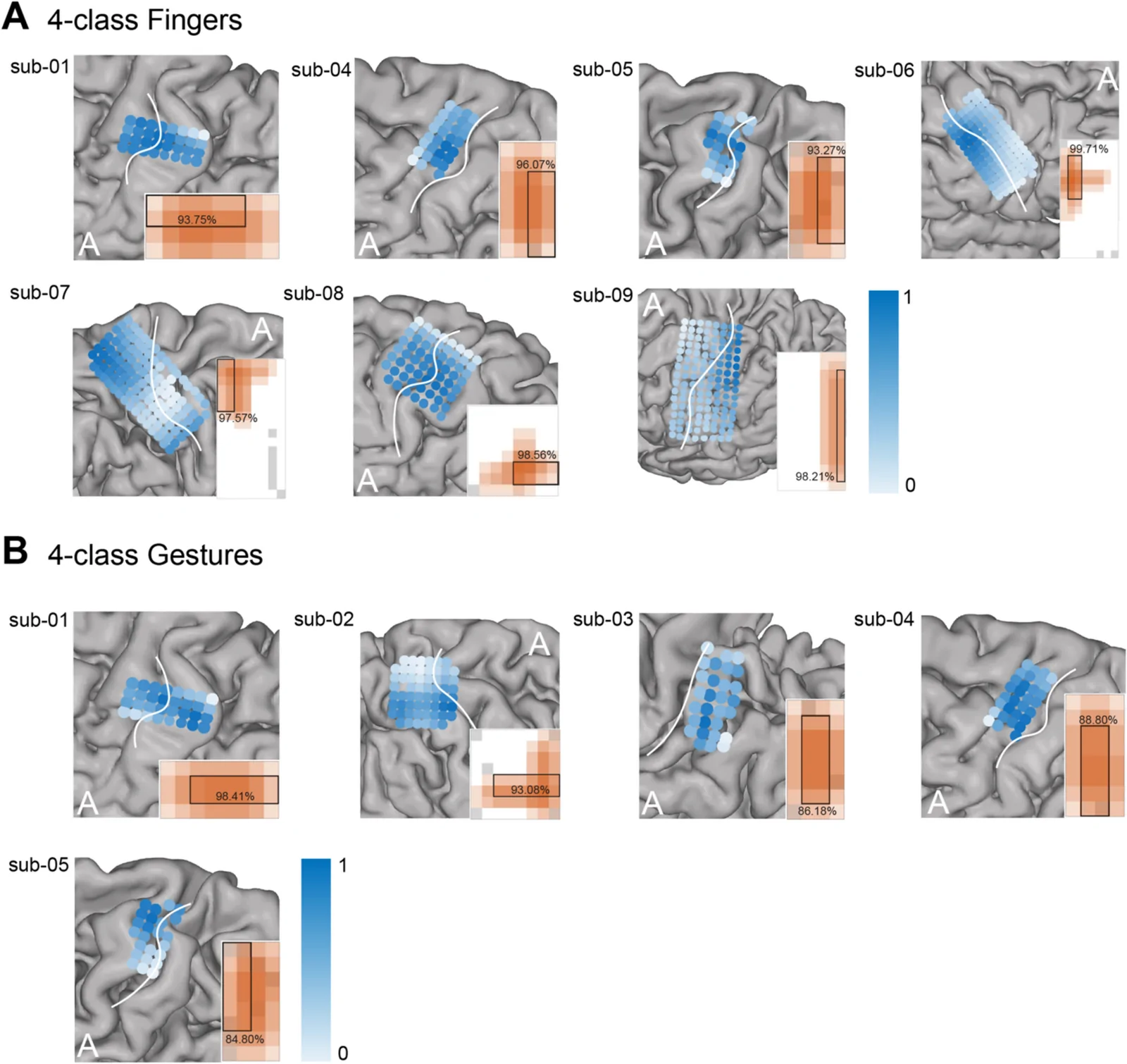
Normalized channel importance and smallest high-performing subgrids. Channel importance for (**A**) the 4-class Finger dataset and (**B**) the 4-class Gesture dataset. MRI reconstruction of the participant’s brain overlaid with a channel importance map representing normalized classification performance (F1 score). The classification importance of a single channel is the normalized average F1 score of all subgrids containing the channel, resulting in a participant-specific range of classification performance from the least (white, 0) to the most informative (blue, 1) channel, indicated with the colorbar. For each participant, the 10 smallest subgrids above the critical point threshold (57mm^2^) are depicted with inserts. When more than 10 subgrids have the same area, the 10 subgrids with the highest F1 score are displayed. Each grid is indicated with a transparent orange area. Darker electrode areas indicate larger overlap between subgrids. The top-performing smallest subgrid of the 10 is marked with a black outline, with its classification performance (F1 score, in %) indicated adjacent to it. Solid white line indicates the central sulcus and A indicates anterior side of the brain

To understand which cortical areas contributed to the discrimination of hand movements, a channel importance map for each participant was computed from the subgrid combinations (see Sect.  2.9). For both the Finger task with 4-class dataset (Fig. 3) and Gesture task with 4-class dataset (Fig. 3B), the most important channels tended to cluster around the hand area. The most important channels were typically located proximal to the central sulcus, often with a single hotspot near the central sulcus or two hotspots along a caudal-rostral axis on M1 and S1, close to the central sulcus. The smallest subgrid per participant and dataset with a surface area larger than 57mm^2^ (Table 3) performed on average as high as the best overall subgrids (best smallest vs. best: 96.6% vs. 98.2% for 4-class Fingers; 90.3% vs. 93.1% for 4-class Gestures; 84.4% vs. 87.8% for 5-class Gestures; 89.85% vs. 92.0% for 8-Class; Wilcoxon signed-rank test, Bonferroni corrected for 4 datasets, ns). Visualization of the 10 smallest subgrids with surface area > 57mm^2^ (Fig. 3 and Supplementary Figure S1) revealed that they were largely rectangular in shape and typically overlapped with highly informative decoding channels.

## Discussion

The advancement of BCI-based assistive and augmentative communication technology towards high-performance decoders appears to coincide with the deployment of larger HD-ECoG grids and increasing electrode counts. These HD-ECoG grids, although effective, involve a large surface area to be covered and a large volume of implanted material. Placement of larger grids typically requires an (extensive) craniotomy associated with an increased risk of (post-)surgical complications and burden for users. In the current work, we demonstrated that HD-ECoG grids can achieve high classification performance with a limited number of optimally placed electrodes. Compared to the originally implanted 32–128 channel grids, the surface area could be substantially reduced without meaningfully sacrificing classifier performance. This reduction was visible also with increasing task complexity from 4- to 8-classes, and suggests that smaller grids could provide equal performance as larger grids for classification problems with relatively limited numbers of classes.

### Smaller Grid Surface Requires Precise Positioning

Our key finding is that for both hand movement tasks, and for a combination of the two, the required surface area for high classification performance converged to a tipping point of around 60mm^2^ cortical coverage. This suggests that a small subset of electrodes, when optimally placed, contains sufficient information for accurate classification of this limited set of hand and/or finger movements. Although research in this area is scarce, demonstrated that reducing the number of high-density channels to just 12 covering the hand area resulted in only a 2% decrease in averaged performance of a click detector in a human clinical trial participant. Furthermore, others have shown that high classification accuracy for four articulator movements can be achieved with high-density grids as small as 1cm^2^ (Salari et al., 2019), and twelve spoken words can be equally well classified from small grids of 32 channels as from larger (> 64 channels) grids when placed optimally on ventral sensorimotor regions (Offenberg et al., 2026).

In our study, we found that electrodes most informative for distinguishing hand movements were clustered near the central sulcus, consistent with previous analyses on ECoG decoding for hand movements (e.g., Siero et al., 2014; Bleichner et al., 2016; Branco et al., 2017; Chestek et al., 2013; Miller et al., 2007; Yanagisawa et al., 2009; Verwoert et al., 2021; Volkova et al., 2019). For several participants, the best subgrids tended to have a rectangular shape with a similar aspect-ratio as the original grid (width x height: 4 × 8, 16 × 8 or 8 × 8). Although the best subgrid represented a considerable decrease in grid area, not all shapes may be equally appropriate for minimally invasive procedures. For example, sub-09 shows a preference for one long strip of 13 electrodes, which likely results from the most informative electrodes being represented in this specific area. Although the cause of this phenomenon remains unclear, these findings suggest that considerable inter-individual variability exists in the spatial organization of informative electrodes.

Interestingly, although the best-performing subgrids largely overlapped with the most informative electrodes, some high-performing subgrids also included channels with relative lower individual decoding importance. These cases suggest electrodes with lower individual relevance may occasionally contribute useful complementary information when combined within a larger ensemble. Even though the 10 smallest subgrids varied in size and shape even within the same participant, the most informative channels for classification were often selected as part of the best subgrid, suggesting that multiple shapes and centers of subgrids can achieve similarly high performance.

Importantly, larger ECoG grids may increase the likelihood of covering the most informative cortical regions for BCI (also extending beyond hand and fingers), thereby reducing reliance on precise positioning, ideal grid geometry, and extensive pre-surgical localization procedures. However, this benefit comes at the cost of a larger surgical footprint and increased invasiveness. In contrast, when smaller grids are employed, surgical footprint and invasiveness are smaller, but optimal placement becomes critical for achieving high BCI performance. Given the established correspondence between ECoG high-frequency band activity and functional magnetic resonance imaging (fMRI) BOLD responses (Siero et al., 2014), pre-surgical fMRI assessments can be highly suitable to determine the optimal target location, as previously demonstrated for hand movements (e.g., Leinders et al., 2023; Metzger et al., 2023; Mitchell et al., 2023).

### Limitations and Future Directions

Our study demonstrated that substantial HD-ECoG surface area reduction compared to 32–128 channel grids can be achieved without a decline in classification performance, with task complexity up to 8-class hand movements. Further research is essential to ascertain whether this trend continues with more complex task designs involving a greater number of classes within the same task or with continuous dimensions. Yet, even if this is not the case, the current results will still be of value for future BCI applications that target highly reliable, basic functionality at minimum levels of invasiveness, for example for target populations unable to accomplish high-dimensional BCI control at desired levels of performance. Of note, in our 8‑class dataset we combined tasks performed at different time points, which could introduce amplitude differences between tasks. However, given the high decoding scores obtained in both the separate and combined fingers‑and‑gestures tasks, this likely indicates no substantial intersession variability.

In this study, we employed a straightforward classification model in an artificial cross-sectional design to establish a proof of principle. The extent to which these findings can be generalized to more complex model designs, particularly in online decoding scenarios within real-life BCI contexts, as opposed to the controlled laboratory environment, remains unknown (Derix et al., 2012). Indeed, our results were obtained from a small sample of able-bodied participants, while the typical target group for BCIs are severely paralyzed individuals who lack sensory feedback. Even though the underlying cause of the motor impairment may affect neural signals and the representation of attempted hand movements (Freudenburg et al., 2019; Vansteensel et al., 2024), previous work has indicated that neuroelectrical signal changes associated with attempted movements align well with those of actual movements, despite the lack of sensory feedback (Bruurmijn et al., 2017). This suggests that our current findings hold significance for BCI users who lack sensory feedback of their movement attempts.

Lastly, we underscore the importance of HD electrode grids for accurate classification, as the hotspots for decoding hand movements can be relatively small. To tailor HD-ECoG grids to such small areas, the electrode spacing could be further reduced to an inter-electrode distance of 1 mm, which has been shown to effectively capture neural signals (Slutzky et al., 2010; Geukes et al., 2024). Our results showed that for specific tasks, such as hand movement decoding, extensive coverage is not necessary to allow high classification performance of a limited number of classes, suggesting that precisely localized high-density ECoG grids (Viventi et al., 2011; Khodagholy et al., 2011, 2015; Vomero et al., 2020; Hettick et al., 2022) could be a promising future direction for combining maximum implant safety with high accuracy classification in multi-degree control settings, given a small volume of implanted material and the possibility of a small size skull opening.

## Conclusion

The present study demonstrates that small high-density ECoG grids can achieve high classification performance of discrete hand movements when placed optimally over the sensorimotor hand region. Our findings indicate that surface area reductions of up to 94% (compared to a 128-channel grid) are feasible without compromising performance. Across the participants and task configurations studied, classification performance for decoding individual fingers and gestures typically remained near full-grid levels with declining subgrid surface area, until the surface area dropped below a conservative threshold of approximately 60mm^2^ of cortical coverage. These results suggest that substantial reductions in redundant surface area could advance scalability of ECoG-based BCIs by enabling less invasive surgical procedures and reducing post-operative risks, while still enabling multi degrees-of-freedom BCI functionality, paving the way for usable, accessible and safe BCI implementations in clinical settings.

## Supplementary Information

Below is the link to the electronic supplementary material.

Supplementary Material 1

## Data Availability

The data that support the findings of this study are not publicly available due to privacy restrictions. However, data can be provided upon reasonable request and subject to legal data transfer agreement.

## Code Availability

The code describing the subgrid search algorithm (SpatialExhaustiveSearch.py), which evaluates all possible subgrid combinations within the ECoG grid, is available in GitHub upon publication. https://github.com/UMCU-RIBS/Subgrid-Optimization/.
