# Supplementary material for: Optimal Size of Electrocorticography Grids for Classification of Hand Movements
Source: Neuroinformatics. 2026 Aug 7;24(3):52. doi: 10.1007/s12021-026-09807-z (PMC13447543; doi:10.1007/s12021-026-09807-z)
Supplement: Supplementary file 1 [file 12021_2026_9807_MOESM1_ESM.docx]

**Supplementary Materials**

**Supplementary Figure S1. Normalized channel importance and smallest high-performing subgrids**. Channel importance for (A) the 5-class Gesture dataset and (B) the 8-class dataset. MRI reconstruction of the participant’s brain overlaid with a channel importance map representing normalized classification performance (F1 score). The classification importance of a single channel is the normalized average F1 score of all subgrids containing the channel, resulting in a participant-specific range of classification performance from the least (white, 0) to the most informative (blue, 1) channel, indicated with the colorbar. For each participant, the 10 smallest subgrids above the critical point threshold (57mm^2^) with highest F1 score are depicted with inserts. Each grid is indicated with a transparent orange area. Darker electrodes aeras indicate larger overlap between subgrids. The top-performing smallest subgrid of the 10 is marked with a black outline, with its classification performance (F1 score, in %) indicated adjacent to it. Solid white line indicates the central sulcus and A indicates anterior side of the brain.
